# In the Spotlight—Established Researcher

**DOI:** 10.1002/jez.b.23310

**Published:** 2025-07-03

**Authors:** Gregor Bucher

**Affiliations:** ^1^ Department of Evolutionary Developmental Genetics Georg‐August‐University Göttingen Germany


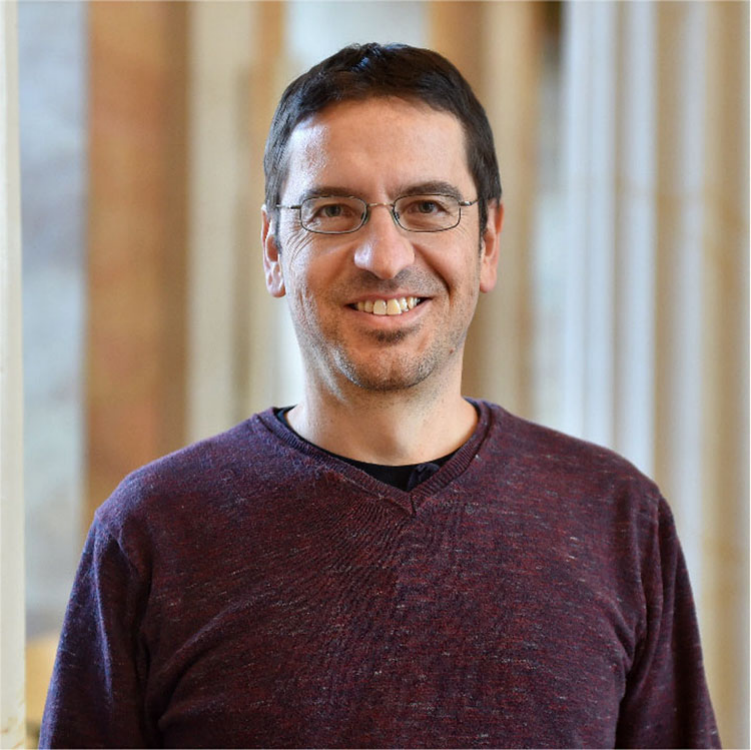


Gregor Bucher was awarded the Heisenberg Professorship of the German Science Foundation (DFG) in 2013 and since 2017 has been Full Professor at the University of Göttingen. He has been interested in the genetic basis of the development and evolution of the insect head and brain and has contributed to using RNAi in pest control. Further, his lab has been pioneering transgenic tools for the emerging model organism *Tribolium Castaneum*, the red flour beetle. He initiated and led the first genome‐wide RNAi screen in an insect outside *Drosophila* (DFG research unit FOR1234 “*iBeetle*”) and has been part of the steering committee of the DFG priority program *SPP2349 GEvol*.

Gregor Bucher is a Guest Coeditor of this special issue on the *Genomic Basis of Evolutionary Innovations in Insect*s.

Google Scholar page: https://scholar.google.com/citations?user=rRpyk9sAAAAJ

## 1 With Whom and Where Did You Study?

I did my studies in genetics, zoology, and medical physiology at the Ludwig‐Maximilians‐University (LMU) in Munich. I especially enjoyed the lectures and courses on developmental biology and still build on the concepts so clearly presented by Harry MacWilliams. The course, which I most vividly remember was on hydra regeneration by Charles David and Thomas Bosch: Even after dissociating these little creatures into single cells and pelleting those cells via centrifugation, they would eventually start growing out little heads.

## 2 What Got You Interested in Biology? When Did You Know Evo Devo Was for You?

Understanding how things actually function has always been interesting to me—starting from the mechanics of my cassette recorder to the formation of thunderstorm clouds. But understanding life has always been most fascinating to me; so I had contemplated several closely related study programs: chemistry (with a focus on biochemistry) or medicine (with a focus on biomedical research) ending up with biology.

Funded by DAAD, I went to study in Concepción, Chile for 1 year. There I took classes on evolution, where we read Darwin's *On the Origin of Species* and at the same time, we observed early development in marine animals in a developmental biology course. This combination made me think that you need to understand the divergence of developmental processes to understand the emergence of morphological diversity. Back in Munich, I looked for research groups in that area and was very, very lucky: At LMU, Diethard Tautz had pioneered the beetle *Tribolium castaneum* as an insect model for EvoDevo research and Martin Klingler had started a mutagenesis screen in this species because, at the time, this tedious genetic procedure had been the only way to gain information on gene function (no RNAi, no genome editing, no transgenesis). So I joined Martin Klingler's lab to follow that approach. One year into my PhD, RNAi was discovered and made this approach (and 1 year of my PhD) obsolete.

## 3 Which Achievement Are You Most Proud Of?

For a long time, EvoDevo research relied on the candidate gene approach—testing the function of orthologs of genes already known from flies or other model organisms to be involved in specific developmental processes. However, once most candidate genes had been studied, it became clear to the community that some important beetle‐specific components were missing. Moreover, for developmental processes not previously studied in flies, there were no candidate gene lists to begin with.

To address these blind spots inherent to the candidate gene approach, we conducted an unbiased, genome‐wide RNAi screen in *Tribolium* in collaboration with several German research groups (Hakeemi et al. 2022; Schmitt‐Engel et al. 2015). Indeed, our *iBeetle* screen uncovered many novel gene functions that were unexpected based on existing fly knowledge. In fact, 40% of the genes we found to produce interesting phenotypes in *Tribolium* had never before been associated with the corresponding developmental processes (Hakeemi et al. 2022).

For example, we identified a large number of genes essential for muscle development in beetles which were not required in flies (Schultheis et al. 2019), as well as a comprehensive set of genes required for the production of defensive secretions—a process absent in flies (Lehmann et al. 2022). Our lab became particularly interested in two genes that were crucial for axis formation in beetles but not in flies. Knocking these genes down caused the larvae to develop a second abdomen in place of the head (Ansari et al. 2018). This striking phenotype revealed that the gene set determining the anterior end of the embryo in beetles is markedly different from that in flies and could not have been revealed using candidate genes. It also showed that axis formation in beetles involves both maternal and zygotic contributions, unlike in flies, where it is controlled exclusively by maternal factors.

With our large‐scale screening platform in place, we were also able to contribute to improving RNAi‐mediated pest control. Previously, a broad and diverse set of genes—often inspired by traditional pesticide targets—had been used. However, our genome‐wide comparisons indicated that the proteasome and other core cellular components were more effective targets (Buer et al. 2025; Ulrich et al. 2015). In fact, the first commercial sprayable RNAi pesticide utilized one of the genes we had identified. While screening for lethality at scale may conceptually not be rocket science, but the application of those results to real‐world pest control made the effort especially rewarding.

## 4 What Is Your Favorite Paper?

One paper I always include in seminars for developmental biology students is by Clark and Akam (2016) at the University of Cambridge. This study demonstrates how questioning a prevailing paradigm can lead to new discoveries, even in areas where the scientific community believed the textbook explanations were complete.

Specifically, by carefully reanalyzing data that had actually been publicly available for years, the authors discovered that a key *timing* component in pair‐rule regulation was likely missing. Building on these analyses, they predicted the timing, expression pattern, and phenotype of this missing gene—and then identified it. Interestingly, all the information about the gene odd‐paired had already been published. It was known that its properties didn't fit the established model for pair‐rule genes, yet its function had not been further investigated. Clark and Akam (2016) revisited this inconsistency and uncovered its novel role in the *timing* of the pair‐rule network.
